# Secondary Syphilis in the Spotlight: Atypical Cutaneous Manifestation Overshadowing Kaposi Sarcoma in a Newly Diagnosed HIV Patient

**DOI:** 10.7759/cureus.72756

**Published:** 2024-10-31

**Authors:** Ricardo A López Pérez, Victoria Sauza Gonzalez, Victor D Acuña Rocha, Anette Fischer Rouyer, Ashley Lilian Villapudua Torres, Rodolfo Franco Márquez

**Affiliations:** 1 Internal Medicine, Hospital Universitario "Dr. José Eleuterio González", Monterrey, MEX; 2 Internal Medicine, Hospital Universitario “Dr. José Eleuterio González”, Monterrey, MEX; 3 Pathology, Hospital Universitario "Dr. José Eleuterio González", Monterrey, MEX

**Keywords:** aids (acquired immunodeficiency syndrome), dermatosis, hiv aids, secondaryism, syphilis

## Abstract

Syphilis, caused by the spirochete *Treponema pallidum*, is a sexually transmitted infection (STI) that has seen a resurgence worldwide, particularly among populations at a higher risk of co-infection with human immunodeficiency virus (HIV). The disease typically progresses through distinct stages: primary, secondary, latent, and tertiary, each with specific clinical manifestations. Secondary syphilis is characterized by systemic involvement and various mucocutaneous symptoms, including a maculopapular rash that frequently involves the palms and soles along with fever, lymphadenopathy, and mucous membrane lesions. However, in patients with HIV co-infection, syphilis may present atypically. The immunosuppression caused by HIV can lead to more severe, atypical, and persistent manifestations of secondary syphilis. Furthermore, the cutaneous features may deviate from the classic presentation, making diagnosis challenging. We report the case of a male in his third decade of life, recently diagnosed with HIV, who presented with diffuse hyperpigmented dermatosis. The unusual presentation, including well-defined brown macules with a generalized distribution, initially raised suspicion for Kaposi’s sarcoma (KS), a frequent cutaneous malignancy seen in HIV patients. Skin biopsy showed a dense perivascular and interstitial inflammatory infiltrate with marked endothelial swelling and vascular proliferation. Immunohistochemistry confirmed the presence of *Treponema* spirochetes, and a positive Venereal Disease Research Laboratory (VDRL) test further supported the diagnosis of secondary syphilis in the context of HIV. Our case underscores the importance of considering secondary syphilis in the differential diagnosis in cases of generalized hyperpigmented dermatosis in newly diagnosed HIV patients, where common conditions such as Kaposi’s sarcoma may obscure the underlying etiology.

## Introduction

Syphilis, caused by the spirochete *Treponema pallidum*, is a sexually transmitted infection with systemic implications, primarily affecting the skin and mucous membranes. It progresses through three distinct stages: primary, secondary, and tertiary. Secondary syphilis, in particular, presents a broad spectrum of clinical manifestations. The characteristic rash can appear as macules or papules, often desquamating, typically involving the palms and soles, and is generally non-pruritic [1,2].

Other common features include oral ulcers, mucous patches, diffuse alopecia, generalized lymphadenopathy, and in moist areas such as the axillae and perineum, soft, raised lesions known as condylomata lata. Secondary syphilis may also involve other organ systems, causing hepatitis, nephritis, and central nervous system involvement [3].

In individuals living with HIV, secondary syphilis is more prevalent and frequently presents with atypical and more severe manifestations, necessitating increased clinical vigilance [4]. Cutaneous manifestations are among the most common presentations and can exhibit unusual characteristics, complicating diagnosis [5-9]. 

Here, we present the case of a male in his third decade of life, without a known history of HIV, who presented with a generalized dermatosis of brown macules. Our case highlights the critical need to consider secondary syphilis in the differential diagnosis of generalized hyperpigmented dermatosis in newly diagnosed HIV patients, where common conditions like Kaposi’s sarcoma can obscure the true etiology.

## Case presentation

**Figure 1:**
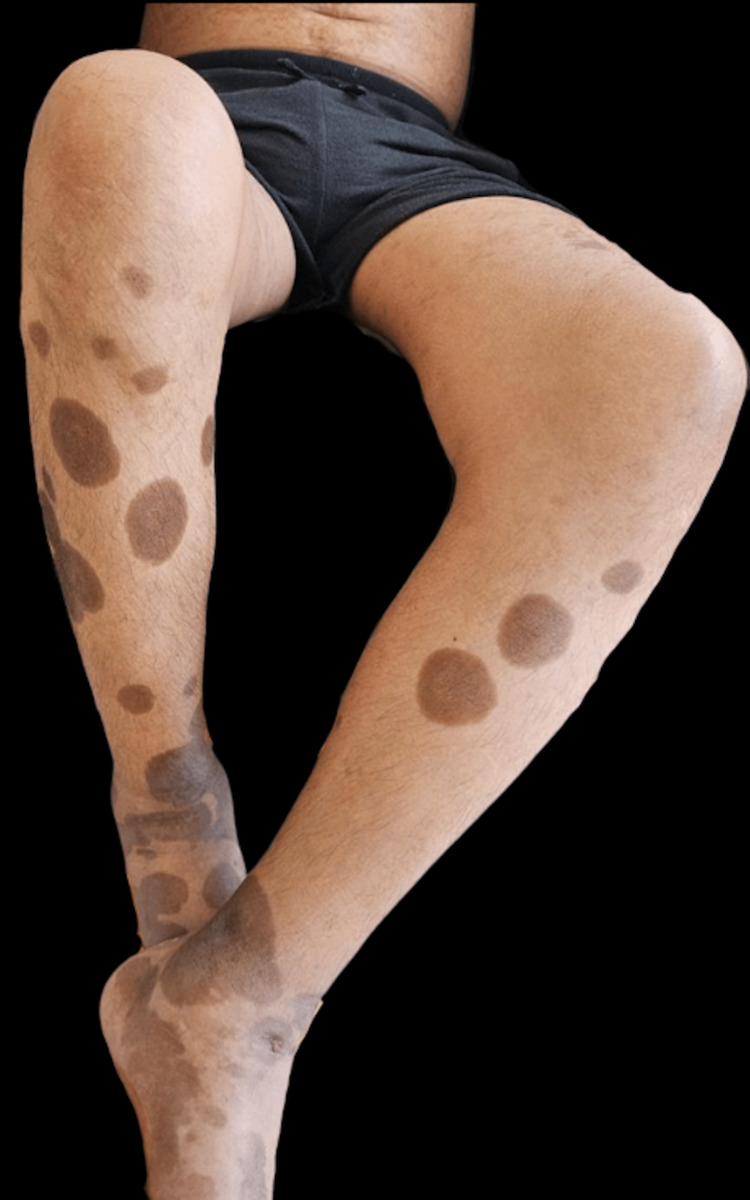
Asymmetrical hyperpigmented dermatosis on the lower extremities Asymmetrical, well-defined brown macules are visible on both lower extremities.

**Figure 2:**
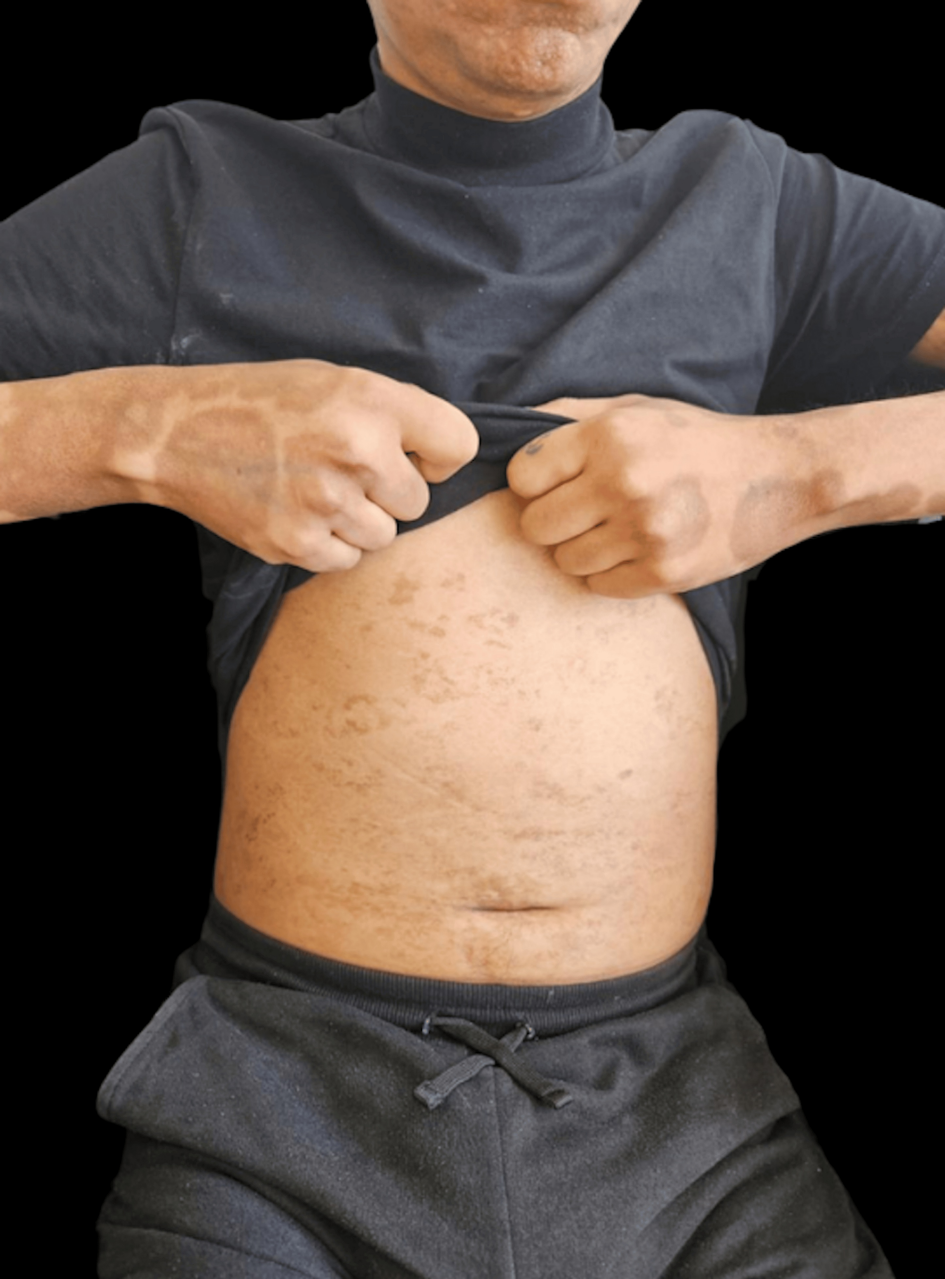
Hyperpigmented dermatosis on the trunk Asymmetrical, well-defined, hyperpigmented brown macules on the trunk

A 37-year-old male with no known history of HIV or other comorbidities, and a history of male-to-male sexual contact, presented to the outpatient dermatology clinic with a generalized dermatosis characterized by symmetrical, well-defined, hyperpigmented brown macules on his hands and palms, which later spread to his lower extremities, trunk, and face (Figures 1, 2). We performed a skin biopsy; however, the patient was lost to follow-up.

**Figure 3:**
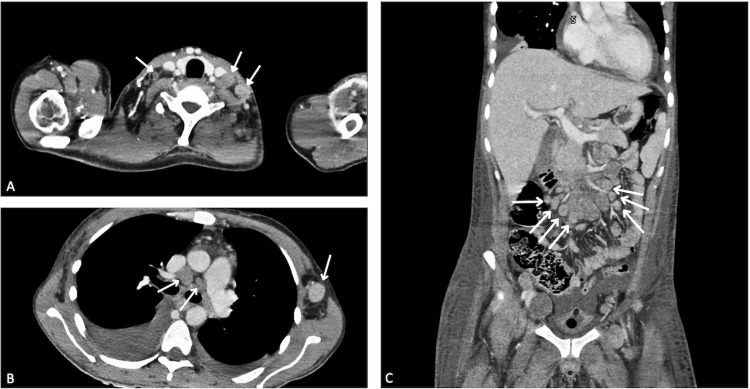
Contrast-enhanced computed tomography (CT) scan of the chest and abdomen Contrast-enhanced CT scan showing A and B: axial sections Arrows: cervical and mediastinal/axillary lymphadenopathy involvement. C: coronal section. Arrows: retroperitoneal lymphadenopathy

Five months later, the patient returned to the emergency department with unintentional weight loss exceeding 15% of his body weight, along with persistent fatigue and generalized weakness. Physical examination revealed mobile, non-tender cervical and axillary lymphadenopathy. Vital signs upon arrival were: blood pressure 120/70 mmHg, heart rate 127 bpm, respiratory rate 18 breaths per minute, and temperature 36.7 °C. Initial laboratory tests are detailed in Table 1. We performed a contrast-enhanced computed tomography (CT) scan of the chest and abdomen due to the presence of lymphadenopathy, which revealed cervical, mediastinal, axillary, retroperitoneal, and inguinal lymphadenopathy, as well as bilateral pleural effusion with right-sided predominance (Figure 3).

**Table 1:** Initial laboratory results for the patient at admission

Category	Result			Normal range
Blood Counts				
Hemoglobin (mg/dL)	6.65			12.1-15.1
Hematocrit (%)	22.1			36.1-44.3
White Blood Count (K/mcL)	14.2			12.9 4.5-11.0
Neutrophils (K/mcL)	10.9			11.5 1.8-7.8
Lymphocytes (K/mcL)	1.9			0.6-3.4
Metabolic Panel				
Platelets (K/mcL)	45.3		150-450	
Creatinine (mg/dL)	0.6		0.59-1.04	
Blood Urea Nitrogen (mg/dL)	13		35 7-20	
Glucose (mg/dL)	116		74-106	
Albumin (g/dL)	1.9		3.4-5.4	
Inflammatory Markers				
Erythrocyte Sedimentation Rate (mm/hr)	32	0-29		
C-Reactive Protein (mg/dL)	9.9	<1.0		
Hepatic Function				
Total Bilirubin (mg/dL)	0.6	0.2-1.0		
Aspartate Aminotransferase (UI/L)	20	5-40		
Alanine Aminotransferase (UI/L)	6	7-56		
Lactate Dehydrogenase (UI/L)	275	91-180		

HIV testing was performed, revealing positive HIV-1 and HIV-2 antibody tests, with a subsequent viral load of 38,448 copies/mL and a CD4 count of 399 cells/µL. Further testing for opportunistic infections, including serum Cryptococcal antigen and an interferon-gamma release assay for tuberculosis, was conducted, both of which were negative. The serum Venereal Disease Research Laboratory (VDRL) test for syphilis was positive at a titer of 1:32.

**Figure 4:**
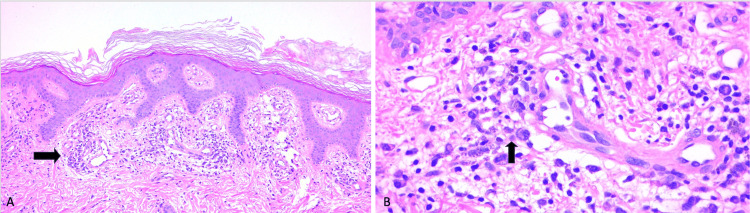
Histological section of the skin from the trunk A) Arrow: perivascular inflammatory process in the superficial dermis, magnification 20x; B) Arrow: lymphoplasmacytic infiltrate around blood vessels, magnification 40x

**Figure 5:**
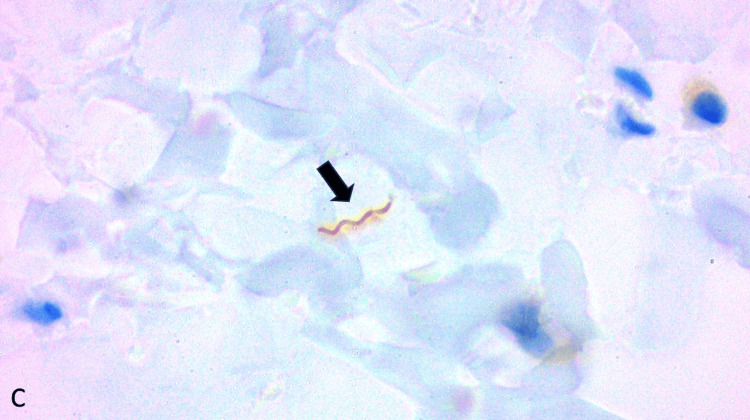
Immunohistochemical staining showing positive reactivity for Treponema Arrow: Positive Treponema stain for structures compatible with spirochetes, magnification 100x

The skin biopsy revealed a dense perivascular and interstitial inflammatory infiltrate composed of lymphocytes, plasma cells, and histiocytes (Figure 4). There was marked endothelial swelling and prominent vascular proliferation. Warthin-Starry staining was negative; therefore, we performed immunohistochemical staining for Treponema, which was positive for the presence of spirochetes (Figure 5), confirming a diagnosis of secondary syphilis, particularly concerning in the context of concurrent HIV infection.

**Figure 6:**
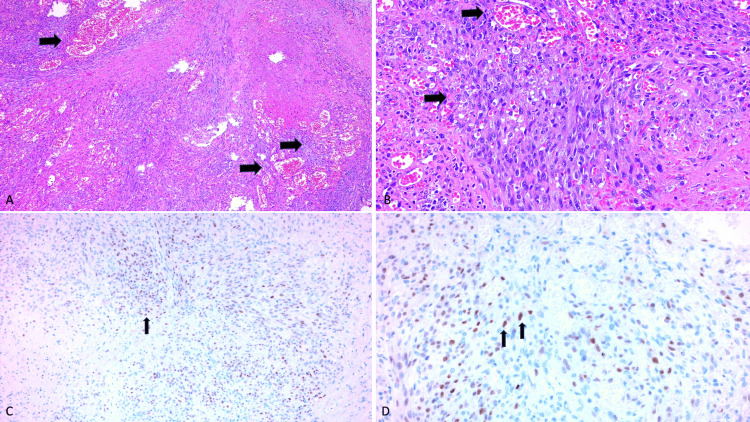
Histological section of a cervical lymph node A and B) Lymph node biopsy showing: Arrows: Proliferation of dilated vascular channels with round spindle cells, magnification 20x and 40x, respectively. C and D) Immunohistochemistry staining for HHV8 showing: Arrows: granular nuclear positivity, magnification 20x and 40x, respectively.

Given the significant lymphadenopathy, a cervical lymph node biopsy was performed. Histopathological examination demonstrated a dense proliferation of spindle cells with irregular, slit-like vascular spaces, extravasation of erythrocytes, hemosiderin deposits, and a mixed inflammatory infiltrate, consistent with Kaposi sarcoma (Figures 6A, 6B). Immunohistochemical staining for human herpesvirus 8 (HHV-8) was positive, confirming the diagnosis (Figures 6C, 6D).

The patient was initiated on prophylactic treatment with trimethoprim/sulfamethoxazole and benzathine penicillin G for secondary syphilis (2.4 million IU, single dose). During his hospitalization, the patient developed episodes of hematochezia, prompting a colonoscopy. The examination revealed multiple violaceous nodules and plaques throughout the colonic mucosa, predominantly in the descending and sigmoid colon. The lesions varied in size, with some appearing as polypoids and others as flat plaques. Several areas showed friable mucosa with scattered ulcerations, consistent with extensive involvement by Kaposi sarcoma, likely contributing to the ongoing bleeding.

Despite initial management, the patient’s clinical condition deteriorated rapidly, and he developed signs of hypovolemic shock, including hypotension and tachycardia, due to continued gastrointestinal bleeding. Intensive resuscitation efforts, including fluid and blood product replacement, were initiated, but unfortunately, the bleeding persisted and the patient did not respond to treatment. His hemodynamic status continued to worsen, and despite exhaustive efforts, he ultimately passed away due to the severity of his condition.

## Discussion

The clinical presentation of syphilis in patients living with HIV may be atypical due to the state of immunosuppression. We present a patient in whom the initial suspected diagnosis was Kaposi's sarcoma (KS) due to the characteristics of the dermatosis, which was marked by hyperpigmented, brown-colored spots with hyperkeratosis and a generalized distribution, in contrast to the classic presentation of secondary syphilis (a pink-reddish maculopapular rash). Moreover, considering the general symptoms, the lymph node biopsy result, and the nature of the dermatosis, the clinical picture was initially consistent with KS.

However, based on the skin biopsy and VDRL results, cutaneous KS was ruled out. Unlike the majority of reported cases of secondary syphilis in patients with HIV co-infection [5,10-12], our patient did not present with nodular or ulcerated lesions, as previously reported. To our knowledge, there are no other reported cases where secondary syphilis mimicked macular KS. While there have been cases [13-15] of secondary syphilis mimicking KS, their clinical presentations included indurated nodular lesions, similar to other cases of secondary syphilis in HIV co-infection. Furthermore, it is important to highlight that in our patient, secondary syphilis was the primary manifestation of AIDS.

Doung et al. reported the case of an HIV-positive patient with a clinical scenario similar to ours, including cutaneous manifestations, cervical lymphadenopathy, and intestinal bleeding. They described macular lesions with scaling on both hands and isolated black-violaceous papules with necrotic centers on the abdomen, measuring less than 5 cm [9]. However, unlike our patient, these were not generalized lesions, and the cutaneous manifestation was not the main reason for consultation.

A case report published by Gori et al. closely resembles ours, describing a 38-year-old patient with no significant medical history, diagnosed with secondary syphilis co-infected with HIV and Kaposi's sarcoma [13]. Although the lesions differed from those of our patient, the location was similar. Their patient presented with smooth, fixed, reddish-purple papulonodular lesions on the face, arms, trunk, and legs, unlike the hyperpigmented macules observed in our case, which included the hands and palms. Despite a similar diagnostic workup, our patient only presented with fatigue and generalized weakness, without symptoms such as night sweats, abdominal pain, or bloody diarrhea. The VDRL titer in Gori’s case was 1:64, and the CD4+ cell count was 525 cells/µL.

In our case, secondary syphilis was confirmed through biopsy and VDRL results. The patient received appropriate treatment for syphilis, though antiretroviral therapy was delayed. This case emphasizes the importance of conducting a thorough differential diagnosis in HIV patients with dermatitis. Although Kaposi's sarcoma is a common cause of skin lesions in these patients, other etiologies, such as sexually transmitted infections, should not be ruled out.

The management of secondary syphilis in HIV patients requires close monitoring and adequate treatment to prevent long-term complications. Penicillin remains the treatment of choice; however, in patients with HIV infection, higher doses or longer treatments may be necessary due to suboptimal responses associated with probable resistance and immunosuppression, leading to an increased bacterial load [5]. In our patient, it was not possible to assess the response to a single dose of benzathine G penicillin as recommended by European Guidelines [16]; however, an alternative treatment regimen has proven effective in other cases of malignant syphilis in immunosuppressed patients. For example, Lopez et al. reported a 29-year-old woman with a history of hypothyroidism and malnutrition who presented with malignant syphilis. In this case, weekly doses of benzathine G penicillin for three weeks resulted in the resolution of cutaneous lesions, with serological improvement observed after three months [17].

## Conclusions

This case highlights the need for a comprehensive diagnostic approach in patients with HIV and dermatosis. It underscores the importance of conducting a thorough differential diagnosis in HIV patients presenting with skin lesions. While Kaposi's sarcoma is a common cause of skin lesions in these patients, other etiologies, such as sexually transmitted infections, should not be overlooked. The coexistence of secondary syphilis and HIV illustrates the complexity of managing these co-infections and emphasizes the need for timely and accurate diagnosis to provide targeted treatment and improve patient outcomes.
